# Mechanisms of Action and Cell Death Associated with *Clostridium perfringens* Toxins

**DOI:** 10.3390/toxins10050212

**Published:** 2018-05-22

**Authors:** Mauricio A. Navarro, Bruce A. McClane, Francisco A. Uzal

**Affiliations:** 1 California Animal Health and Food Safety Laboratory, San Bernardino Branch, School of Veterinary Medicine, University of California-Davis, San Bernardino, CA 92408, USA; mnavarrob@ucdavis.edu; 2 Department of Microbiology and Molecular Genetics, University of Pittsburgh School of Medicine, Room 420, Bridgeside Point II Building, 450 Technology Drive, Pittsburgh, PA 15219, USA; bamcc@pitt.edu

**Keywords:** *Clostridium perfringens*, toxins, mechanisms, cell death, apoptosis, necrosis

## Abstract

*Clostridium perfringens* uses its large arsenal of protein toxins to produce histotoxic, neurologic and intestinal infections in humans and animals. The major toxins involved in diseases are alpha (CPA), beta (CPB), epsilon (ETX), iota (ITX), enterotoxin (CPE), and necrotic B-like (NetB) toxins. CPA is the main virulence factor involved in gas gangrene in humans, whereas its role in animal diseases is limited and controversial. CPB is responsible for necrotizing enteritis and enterotoxemia, mostly in neonatal individuals of many animal species, including humans. ETX is the main toxin involved in enterotoxemia of sheep and goats. ITX has been implicated in cases of enteritis in rabbits and other animal species; however, its specific role in causing disease has not been proved. CPE is responsible for human food-poisoning and non-foodborne *C. perfringens*-mediated diarrhea. NetB is the cause of necrotic enteritis in chickens. In most cases, host–toxin interaction starts on the plasma membrane of target cells via specific receptors, resulting in the activation of intracellular pathways with a variety of effects, commonly including cell death. In general, the molecular mechanisms of cell death associated with *C. perfringens* toxins involve features of apoptosis, necrosis and/or necroptosis.

## 1. Introduction

The ability of *Clostridium perfringens* to produce a large repertoire of toxins makes it a serious pathogen of humans and animals, being able to produce histotoxic, enteric and/or enterotoxemic diseases [1,2]. Toxin production varies significantly among *C. perfringens* strains, which is the basis for a classification system that was traditionally based upon the presence of encoding genes for alpha (CPA), beta (CPB), epsilon (ETX) and iota (ITX) toxins [1]. This typing system, however, was recently revised to include two additional types, i.e., *C. perfringens* type F strains producing enterotoxin (CPE) but not CPB, ETX or ITX, and *C. perfringens* type G strains producing necrotic enteritis B-like toxin (NetB) [3] (Table 1).

The cellular action of many *C. perfringens* toxins involves initial binding to a receptor located on the plasma membrane of target cells, followed by activation of intracellular pathways and a variety of cytopathic effects that eventually lead to cell death [1,4]. Recent studies of pathogen-induced host cell death have revealed a variety of complex mechanisms involved in this outcome, once believed to be simple and merely incidental [5,6,7]. Beyond the classic concepts of necrosis (an accidental and uncontrolled type of cell death) and apoptosis (a programmed mechanism of cell death), it is now well known that many pathogens are capable of triggering several other pathways which have been extensively reviewed [8,9,10,11]. These include necroptosis (or “programed necrosis”), autophagy, pyroptosis, anoikis, and several others. Understanding the mechanisms of action and factors needed for a pathogen to kill host cells is critical to deciphering bacterial pathogenesis, and it represents a key piece of knowledge to explore novel therapeutic approaches. Here we review the current knowledge on the mechanisms of action of *C. perfringens* toxins, with special emphasis on their effects on cell death.

## 2. *C. perfringens* Alpha Toxin (CPA)

### 2.1. Toxin Genetics, Structure and Role in Disease

The gene encoding CPA is situated in a stable region within the bacterial chromosome and it is present in all *C. perfringens* isolates [1,12]. CPA is a zinc metalloenzyme composed of 370 amino acids, which binds to host cell membranes in the presence of calcium ions [13]. It is divided into two main domains: the catalytic N-domain and the membrane binding C-domain [13,14]. Only the latter is immunoprotective [1]. CPA also has a central loop domain containing a ganglioside (GM1a) binding site [14].

The lethal, hemolytic, and dermonecrotic CPA is the most important virulence factor involved in human gas gangrene (clostridial myonecrosis) [15] which is characterized by necrosis of skeletal muscle, subcutaneous edema and emphysema, shock, multiple organ failure and death [16,17]. In addition, a member of the cholesterol-dependent cytolysin family of pore-forming toxins, named Perfringolysin O (PFO) [18], has been shown to act synergistically with CPA to produce the pathological effects observed in gas gangrene [19]. The role of PFO by itself in the disease, however, seems to be minor [1]. Although *C. perfringens* type A has been associated with cases of gas gangrene in animals, the role of CPA in these species has not been established [4].

### 2.2. General Mechanism of Action

**Figure 1:**
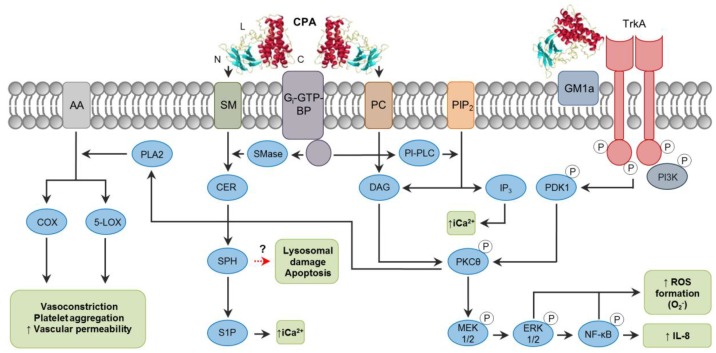
Intracellular pathways involved in *C. perfringens* alpha-toxin (CPA) intracellular action. Note the binding C-domain (C), catalytic N-domain (N) and ganglioside-binding loop domain (L) of CPA (PDB ID: 1CA1). CPA directly hydrolyzes phosphatidylcholine (PC) and sphingomyelin (SM) present in the plasma membrane of target cells. CPA can also activate G_i_-type GTP-binding protein (G_i_-GTP-BP) present in the plasma membrane, which in turn will activate endogenous phospholipases (PI-PLC) and sphingomyelinases (SMase). Phospholipase activity results in the formation of diacylglycerol (DAG), and inositol trisphosphate (IP_3_); the latter mobilizes and increases intracytoplasmic calcium ions (iCa^2+^). Sphingomyelinase action results in ceramide (CER), sphingosine (SPH) and sphingosine-1-phosphate (S1P) formation. In addition, interaction of CPA with the TrkA receptor leads to PDK1 and PKCθ phosphorylation, resulting in activation of the MEK/ERK signaling cascade and NF-κB, which is involved in reactive oxygen species (ROS) and IL-8 formation. A dashed red arrow represents what is concluded and/or suggested by the current authors.

The activity of CPA is highly complex, and it varies among cell types due to factors such as the ratio of phosphatidylcholine (PC) to sphingomyelin (SM) in the plasma membrane, and it is also influenced by local toxin concentrations. For these reasons, many different pathways are affected during CPA action (Figure 1). CPA hydrolyzes PC and SM in the plasma membrane, producing diacylglycerol (DAG) and ceramide (CER), respectively [14]. In addition to these activities, CPA can indirectly activate endogenous host enzymes which have similar phospholipase and sphingomyelinase properties through the interaction with G_i_ type GTP-binding proteins (G_i_-GTP-BPs) (Figure 1) [20]. However, CPA action is not limited only to membrane disruption. The ganglioside-binding site of the toxin in the central loop domain promotes the interaction and tethering of tropomyosin receptor kinase A (TrkA) on cell membranes [21], leading to activation of the MEK/ERK pathway [14]. Interestingly, ganglioside-deficient DonQ or GM95 cells are highly sensitive to CPA action [22,23,24]. Gangliosides are glycosphingolipids that may reduce the fluidity of the plasma membrane, resulting in a tighter packing of phospholipids, and are also associated with electrostatic changes affecting the availability of substrates [22,25]. These effects could interfere with the membrane-disrupting activity of CPA, thus protecting cells from cell damage. Sialic acids are important in the structure of gangliosides [26], and their removal by *C. perfringens* sialidases increased cell sensitivity to CPA in vitro and in vivo [22], implying a potential synergism between CPA and sialidases. This synergistic effect of sialidases has also been proved to increase binding/activity of other *C. perfringens* toxins (particularly NanI sialidase) including CPB, CPE and ETX [27,28].

As mentioned before, CPA can bind to and act on membrane PC and SM, in addition to activating G_i_-GTP-BPs, triggering different pathways depending on the cell type involved (Figure 1). For instance, horse erythrocytes are lysed by intrinsic CPA phospholipase activity on PC, and probably also through activation of endogenous phosphatidylinositol-specific phospholipase C (PI-PLC) [29], whereas sheep erythrocytes which contain only traces of PC are affected by CPA mainly by activation of the SM metabolism via GTP-BPs [20,30]. In rabbit neutrophils and DonQ cells, CPA-induced superoxide anion generation occurs through the activation of endogenous phospholipase C (PLC) and phosphorylation of PI3K via TrkA receptor, and further phosphorylation of PDK1, PKCθ, the MEK1/2, ERK1/2 system and NF-κB [31,32]. This pathway is also important in the generation of IL-8 by A549 cells, in addition to the activation of p38 MAPK, which is believed to stabilize IL-8 mRNA [21]. In this regard, the scarcity of neutrophils within gas gangrene-affected tissues and their accumulation on the vascular endothelium could be due, at least in part, to the effect of elevated IL-8 promoting firm adhesion of neutrophils to extracellular matrix proteins [21]. Recent studies have also reported impaired differentiation and replenishment of neutrophils in peripheral blood induced by CPA [33], effects that have been attributed to alterations in GM1a-containing lipid rafts in those cells [34]. Furthermore, the persistence of an anaerobic microenvironment during gas gangrene is believed to be the result of CPA-induced activation of arachidonic acid (AA) metabolism through phospholipase A2 (PLA2), generating prostaglandins, tromboxanes and leukotriens, which are associated with inflammation, muscle contraction, platelet aggregation and vasoconstriction, resulting in decreased perfusion [12].

### 2.3. Mechanisms of Cell Death

Lytic concentrations of CPA can result in extensive degradation of plasma membrane and lactate dehydrogenase (LDH) release [22,35], which is characteristic of necrosis [36]. Sub-lytic concentrations of CPA are associated with activation of the MEK/ERK pathway and generation of reactive oxygen species (ROS) [23], which in certain amounts can lead to oxidative stress in cells and activate intrinsic mechanisms of apoptosis [37]. A recent study [24] suggests that the SM metabolism induced by a low CPA dose is associated with the generation of pro-apoptotic mediators, such as CER, N-acylethanolamine and saturated fatty acids, and the release of mitochondrial cytochrome C, activation of caspase-3 and increased exposure of phosphatidylserine in GM95 cells. The role of CER as a second messenger involved in apoptosis has been previously described [38,39]. CER is processed to sphingosine (SPH) which can accumulate inside of and induce rupture of lysosomes, releasing lysosomal proteases involved in apoptosis [40]. ROS production can also lead to lysosomal damage [41] when CPA is endocytosed through cholesterol-containing caveolae and, within endosomal compartments, it activates signaling pathways along its trafficking routes [32]. High levels of intracytoplasmic Ca^2+^ (iCa^2+^) commonly play a role in pre-lethal events of both apoptosis and necrosis [42,43]. CPA leads to the formation of sphingosine-1-phosphate (S1P) from sphingosine (SPH) [30], and inositol trisphosphate (IP_3_) from PIP_2_ [12] (Figure 1); both S1P and IP_3_ are associated with mobilization and increase of iCa^2+^ [44]. To our knowledge, however, the role of iCa^2+^ as a trigger for specific cell death pathways associated with CPA has not been investigated.

## 3. *C. perfringens* Beta Toxin (CPB)

### 3.1. Toxin Genetics, Structure and Role in Disease

The *cpb* gene is carried on large virulence plasmids which can contain additional toxin genes such as *cpe* or the toxin perfringens Large (*tpeL*) [45,46]. CPB is encoded as a prototoxin of 336 amino acids, including a 27-amino acid signal sequence which is removed during secretion, resulting in a mature toxin of ~35 kDa [47,48]. The deduced amino acid sequence of CPB shares significant homology with delta toxin and beta-pore forming toxins (β-PFTs) of *Staphylococcus aureus* [47]. CPB is classified as a clostridial β-PFT of the α-hemolysin family [49], and it is characterized for being extremely sensitive to trypsin and other proteases [50]. Due to this trypsin sensitivity, CPB is only active in the presence of trypsin inhibitors, both in vitro and in vivo [4]. CPB variants exist that may possess different trypsin sensitivity and in vitro cytotoxic activity [51].

CPB is produced by *C. perfringens* types B and C and it is responsible for diseases in several animal species and humans [1]. Type B isolates have been associated with fatal hemorrhagic dysentery in sheep, whereas type C isolates cause necrotic enteritis and/or enterotoxemias in several livestock species, and enteritis necroticans (Pigbel) in humans [1,4,52]. Studies using isogenic-null mutants demonstrated that CPB is necessary and sufficient to reproduce the intestinal pathology of type C isolates in rabbit intestinal loops [53,54], and in goats, which are natural hosts for *C. perfringens* type C disease [55]. CPB was also shown to be responsible for lethality in a mouse enterotoxemia model [56].

### 3.2. General Mechanism of Action

The pathology of the spontaneous disease associated with CPB is characterized by hemorrhage and necrosis of the epithelium of the small and sometimes large intestine. CPB-associated damage begins in the intestinal mucosa but may progress to all layers of the intestine. Fibrin thrombi occluding the superficial microvasculature present in the lamina propria are characteristic of CPB-associated intestinal disease [4]. Whether the mucosal epithelium is the primary target for the toxin to induce damage or if this is secondary to vascular thrombosis has been a frequent matter of debate. In the rabbit intestinal loop model, inoculation of the wild-type *C. perfringens* type C isolate CN3685 produced necrosis of the villous tip before thrombosis was apparent, suggesting early intestinal epithelial injury [53]. On the other hand, CPB was demonstrated by immunohistochemistry only in endothelial cells within the lamina propria in naturally-occurring cases of necrotic enteritis in piglets [57] and in a human patient [58], as well as in an experimental infection model in pigs associated with early vascular lesions [59]. In all those studies, however, tissue necrosis was already present and CPB binding to epithelial cells was not detected. Further CPB immunohistochemical studies on porcine jejunal explants showed no binding of the toxin to epithelial cells, and the presence of an intact epithelial layer inhibited CPB detection into deeper intestinal layers of the intestine [60]. The same study showed no cytopathic effect in cultured intestinal porcine epithelial cells (IPEC-J2) and primary jejunal epithelial cells when co-incubated with recombinant CPB (rCPB). An initial epithelial damage was proposed for CPB to reach the microvasculature in the lamina propria in the porcine intestinal model. However, since the supernatants of two *C. perfringens* type C strains induced IPEC-J2 cell damage that was not prevented with anti-CPB antibodies, it was concluded that CPB was not responsible for the cytopathic effect observed [60]. In addition, an indirect vascular effect of CPB in mouse skin was observed, in which the toxin induced the release of substance P (SP), an NK_1_ agonist from sensory neurons, leading to the release of TNF-α and plasma extravasation [61,62].

**Figure 2:**
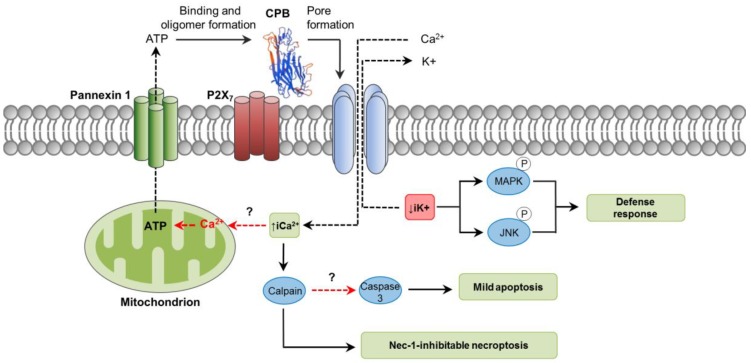
Pathways involved in *C. perfringens* beta-toxin (CPB) intracellular action. CPB structure was predicted by the SwissModel online software (Genbank ID: L13198) using standard settings. Initial binding of CPB to its potential receptor, the ATP-gated P2X_7_ receptor, induces a rapid peak of ATP release from target cells. This ATP loss is not associated with cell lysis and it would occur through the ATP-release channel pannexin 1. The released ATP would stimulate further CPB binding and oligomer formation, facilitating the pore-forming activity of the toxin. Pore formation results in Ca^2+^ influx and loss of intracytoplasmic K^+^ (iK^+^). Increase of iCa^2+^ is associated with calpain activation and necroptosis, which is inhibited by Nec-1; only low levels of caspase-3 activation occurs, suggesting that apoptosis is not a significant mechanism of cell death. Decrease iK^+^ is associated with the activation of MAPK and JNK, which activate host cell survival and defense pathways. Dashed red arrows represent what is concluded and/or suggested by the current authors. A dashed black arrow shows direction of movement.

As an oligomerizing, pore-forming toxin, CPB is capable of forming functional pores in plasma membranes of susceptible cells [63,64]. These pores allow the efflux of K^+^ and the entry of Ca^2+^, Na^+^ and Cl^−^ into the cells, followed by cell swelling [64]. Potassium efflux induced by CPB promotes phosphorylation of p38 MAP and JNK kinases, which activate pathways associated with host cell survival and adaptation [65]. Susceptibility of cells to CPB action has been tested in different human hematopoietic tumor cell lines, with THP-1 and U937 cells showing the highest CPB-induced cytotoxicity compared to HL-60, BALL-1 and MOLT-4 [65]. Using primary porcine aortic endothelial cells, CPB was demonstrated to induce rapid disruption of the actin cytoskeleton, cell border retraction and cell shrinkage [66]. Differences in cell susceptibility clearly suggest that the toxin binds to specific receptors. It has been recently shown that CPB putatively binds to the ATP-gated P2X_7_ receptor, after which ATP is released from the cell through an ATP channel called Pannexin 1 (Panx1) [67,68]. How this ATP release related to CPB binding occurs is not known; however, it is not associated with cell lysis [68]. Perhaps, an initial influx of Ca^2+^ through the CPB pores could induce mitochondrial ATP production and later release from the affected cell [69]. It is presumed that this rapid peak of ATP release from cells through Panx1 could stimulate further CPB oligomer formation and consequent cytotoxicity (Figure 2) [68]. Functional and physical interactions of Panx1 with P2X_7_ receptor bound to CPB would influence Panx1 channels opening promoting ATP release [68]. Both enterocytes and endothelial cells express the P2X_7_ receptor and Panx1 [70,71], but their role in CPB-associated disease in vivo needs further investigation.

### 3.3. Mechanisms of Cell Death

Studies using porcine endothelial cells in vitro have shown that rCPB rapidly induces cellular events consistent with necrosis, including LDH release and propidium iodide (PI) intake [72]. Both events were inhibited by calpain inhibitors and necrostatin-1, a RIP-1 inhibitor, which suggested that CPB-induced necrotic cell death was not a passive event but occurred through a programmed biochemical pathway. The authors concluded their necrostatin effects indicated CPB induces necroptosis. However, it is worth mentioning that RIP-1 (the necrostain target) can also be involved in apoptosis [73]. On the other hand, the referred study detected low levels of caspase-3 activation, but not appreciable DNA fragmentation, suggesting that apoptosis was not an important cell death pathway at the toxin concentrations and times of incubation tested. How caspase-3 activation occurred in those cells was not explored. It may be hypothesized that the rise of iCa^2+^ could play a key role since it has been associated with both apoptosis and necroptosis through the activation of calpains [8,43].

## 4. *C. perfringens* Epsilon Toxin (ETX)

### 4.1. Toxin Genetics, Structure and Role in Disease

The *etx* gene is localized on plasmids, where it can be associated with other toxin genes such as *cpb2* and *cpe* [74]. ETX is secreted as a poorly active protototoxin of ~33 kDa, which is activated by intestinal proteases produced by the host such as trypsin, α-chymotrypsin, carboxypeptidases, and/or sometimes by λ-protease, which is produced by some strains of *C. perfringens* [75,76,77]. Removal of the 13 N-terminal and 29 C-terminal residues by trypsin or α-chymotrypsin results in a mature toxin that is 1000 times more toxic than the prototoxin. *C. perfringens* λ-protease removes only 10 of the N-terminal residues [75,78]. In an ex vivo study using caprine intestinal contents, it was demonstrated that ETX prototoxin is processed in a step-wise fashion into a stable, active ~27 kDa band on SDS-PAGE [79]. The ~27 kDa band was shown to contain three ETX species with varying C-terminal residues. Each of those ETX species was cytotoxic. This additional processing of ETX C-terminal sequences is apparently caused by intestinal carboxypeptidases, based upon inhibitor studies [79]. Those findings suggest that ETX activation in vivo is more complex than previously reported when using only purified proteases [77,79]. This toxin is classified as a heptameric β-PFTs of the aerolysin family given its structural similarity to aerolysin produced by *Aeromonas* sp., although no significant amino acid sequence identity is shared between them [49].

ETX, the third most potent clostridial toxin known after *C. botulinum* and *C. tetani* toxins, is synthesized by strains of *C. perfringens* types B and D. ETX-producing *C. perfringens* type D strains are the most common cause of clostridial enterotoxemia in sheep, goats and less frequently cattle [4,80,81]. Studies using isogenic *C. perfringens etx* -null mutants in sheep, goats and mice models have confirmed that ETX is the main virulence factor responsible for all lesions and symptoms due to *C. perfringens* type D enterotoxemia [82,83]. Because enterotoxemia results from toxin absorption from the gut into the circulation, toxoid vaccines are used to protect livestock against type D infections [84].

### 4.2. General Mechanism of Action

ETX is produced in the intestine, and even though fibrinonecrotic colitis may be present in cases of caprine enterotoxemia, the toxin mainly targets distant organs such as the central nervous system, lungs and heart [4]. It is believed that ETX increases intestinal permeability, hence facilitating its own absorption [4,84]. This effect is not fully understood, but it involves the opening of the mucosa tight junctions and degenerative changes in the lamina propria of the gut [85]. Microscopically, lesions in the brain tend to be multifocal and characterized by perivascular and intramural vascular edema, hemorrhage, and in more chronic cases, necrosis of the white matter and swelling of astrocytes and axons [4]. The latter is commonly symmetrical and bilateral [86]. The origin of these lesions is believed to be the result of an initial binding of the toxin to endothelial cells of the brain–blood barrier (BBB) since intravenous inoculation of ETX labeled with green-fluorescent protein (GFP) in mice revealed binding of the toxin to those cells [87]. After ETX binding, endothelial cells become swollen, vacuolated and necrotic [88]. By disrupting the BBB, leakage of fluids and proteins occurs, leading to an edema that causes mechanical damage and hypoxia of the neural parenchyma [83]. In response to this edema, astrocytes of rats and sheep overexpress aquaporin-4, which is believed to be the host’s attempt to reduce the excess of fluid from perivascular spaces [83,89]. In addition to this indirect effect on the brain, ETX can directly affect certain types of neurons and oligodendrocytes, but not astrocytes [90,91,92]. Mice injected with ETX fused to GFP also revealed severe kidney alterations, including degeneration of distal tubules and hemorrhages in the medulla [93]. However, renal lesions are not usually observed in natural disease and the so-called “pulpy kidney” is believed to be a postmortem change rather than a true lesion [84].

Binding and cytotoxic activities of ETX have been extensively studied using cell lines from renal origin of different species in which spontaneous cases of type D enterotoxemia have not been reported, including dogs (Madin–Darby canine kidney cells, MDCK), mice and humans. However, kidney cell lines from species naturally affected by ETX such as sheep and cattle are resistant [94].

**Figure 3:**
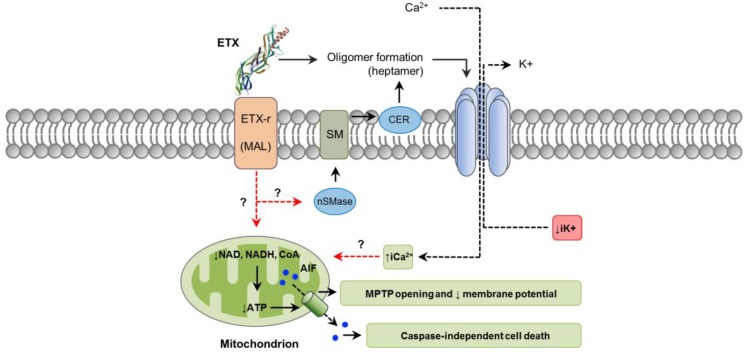
Pathways involved in *C. perfringens* epsilon-toxin (ETX) intracellular action. ETX (PDB ID: 1UYJ) action involves binding to its receptor (ETX-r), such as the myelin and lymphocyte (MAL) protein. Neutral sphingomyelinase (nSMase) may be activated resulting in sphingomyelin (SM) hydrolysis and ceramide (CER) production, which would facilitate ETX oligomer formation. Oligomerization results in a heptameric pore that induces a rapid loss of iK^+^, entry of Cl^−^ and Na^+^ (not shown), followed later by an increase of iCa^2+^. ETX-affected cells also lose important coenzymes required for energy production, including NAD^+^, NADH and CoA, contributing the formation of the mitochondrial permeability transition pore (MPTP). This would facilitate the translocation of the apoptosis-inducing factor (AIF), a caspase independent cell death factor, from mitochondria to the nucleus. Dashed red arrows represent what is concluded and/or suggested by the current authors. A dashed black arrow shows direction of movement.

The initial steps of ETX action involve binding to an unidentified cell surface receptor and heptamerization into a prepore on the membrane surface, followed by insertion into the plasma membrane to form an active pore [95,96,97]. Recently, it was reported that ETX activates neutral sphingomyelinase (nSMase) which in turn facilitates oligomer formation by producing ceramide in the plasma membrane [98]. In fact, knockdown of nSMase blocked oligomer formation and ETX-induced cell death in ACHN cells (derived from human renal adenocarcinoma) (Figure 3) [98]. Since that particular study used ETX isolated from *C. perfringens* culture, it is possible that impurities containing CPA, which also induces CER formation [24], could have been in part responsible for this effect, which might indicate a synergistic effect between these two toxins. This possibility, however, has not been explored. In vitro, ETX binds to the hepatitis A virus cellular receptor (HAVCR1) in MDCK and ACHN cells, facilitating cytotoxicity [99]. Nevertheless, induced expression of HAVCR1 in cells resistant to ETX did not result in sensitivity to the toxin [99]. The myelin and lymphocyte (MAL) protein has been found to be required for ETX cytotoxicity in several cell types [100]. MAL is expressed in many cells known to be targeted by ETX, including endothelial cells, renal cells and oligodendrocytes. Neurons, which have been suggested but not definitively proved to be sensitive to ETX, do not express MAL [101]. It has been hypothesized that MAL may act as a specific receptor for ETX, as a protein involved in assembly of a multi-protein complex required for ETX interaction with the plasma membrane, or even allowing a mechanism independent of pore formation [100,102].

### 4.3. Mechanisms of Cell Death

Mechanisms of cell death and intracellular pathways associated with ETX action are not fully characterized. However, the toxin induces cellular changes that are compatible with necrosis, including initial and marked cell swelling, followed by disappearance of mitochondria, blebbing, membrane disruption, ATP depletion, reduction of nucleus size and increased PI uptake; no DNA fragmentation occurs [81,103,104]. Pore formation in affected cells leads to a rapid loss of intracellular K^+^, entry of Cl^−^ and Na^+^, followed later by an increase of iCa^2+^ [103]. Whether this imbalance of intracellular electrolytes is involved in activating specific downstream signaling events leading to cell death has not been determined, although it seems likely. ETX-affected cells lose important coenzymes critical for energy production, particularly nicotinamide adenine dinucleotide (NAD^+^ and NADH) and coenzyme A, contributing to the dissipation of the mitochondrial membrane potential and opening of the mitochondrial permeability transition pore [104,105]. Consistent with those findings, AMP-activated protein kinase, an intracellular sensor of ATP, is stimulated in ETX-affected cells [104]. In addition, apoptosis-inducing factor (AIF), a potent caspase-independent cell death factor, is translocated from mitochondria to the nucleus (Figure 3) [104]. Paracellular permeability to macromolecules is not significantly increased in renal cells treated with ETX in vitro, and actin cytoskeleton and organization of tight and adherens junctions are not affected [104,106]. In the brain, ETX can bind to certain types of neurons and cause a decrease in electrical membrane resistance [104], which is attributed to pore formation. This induces membrane depolarization associated with an increase of iCa^2+^, followed by the release of the excitatory neurotransmitter, glutamate [92,107]. Interestingly, ETX does not form pores in the plasma membrane of oligodendrocytes [92]; thus, the associated glutamate release, Ca^2+^ fluctuations and demyelination occurs by a different mechanism, involving intracellular pathways occurring as a consequence of ETX–MAL interaction [92,102].

## 5. *C. perfringens* Iota Toxin (ITX)

### 5.1. Toxin Genetics, Structure and Role in Disease

**Figure 4:**
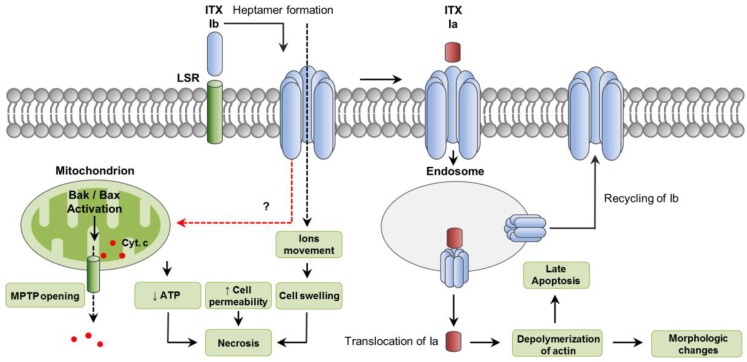
Intracellular pathways involved in *C. perfringens* iota-toxin (ITX) action. ITX is a binary toxin composed of an enzyme component (Ia) and a binding component (Ib). The lipolysis-stimulated lipoprotein receptor (LSR) has been reported a cellular receptor for Ib; after binding, Ib oligomerizes in a heptameric, functional pore that allows the movement of ions, and the translocation of Ia. Cytotoxicity involves features of necrosis, including decreased ATP, increased cell permeability and cell swelling. Bak and Bax activation also occurs leading to cytochrome C release from mitochondria. In addition, both Ia and Ib are internalize in endosomes. While a small proportion of Ib is recycled back to the plasma membrane, Ia is translocated into the cytoplasm from late endosomes. Once in the cytoplasm, Ia exerts its ADP-ribosylating activity, leading to depolymerization of actin which results in morphologic changes. Cytosolic stability of Ia would induce, likely via depolymerization of actin, a delayed caspase-3 activation and apoptosis. A dashed red arrow represents what is concluded and/or suggested by the current authors. A dashed black arrow shows direction of movement.

ITX is a clostridial binary toxin composed of an enzyme component (Ia) and a binding component (Ib) (Figure 4) [108]. Both components are encoded by two separate genes, *iap* and *iab*, which are located on large, potentially conjugative plasmids [109]. Ib is synthetized as an inactive form (100 kDa) which becomes active after proteolytic removal of a 20 kDa N-terminal fragment by trypsin or chymotrypsin [110].

ITX is produced by *C. perfringens* type E strains. Enteric disease associated with this toxinotype has been suggested in many animal species. However, the diagnosis in most of those cases was based only on isolation of *C. perfringens* type E from intestinal content of animals with hemorrhagic enteritis, which is not considered to be a diagnostic criterion for *C. perfringens* type E infection. In addition, Koch’s molecular postulates have not been fulfilled for type E disease. The specific role of ITX in disease remains, therefore, unclear [111]. In the past, cases of enterotoxemia in rabbits were attributed to *C. perfringens* type E, based on the detection of ITX in the intestine of affected animals. Nevertheless, it has been hypothesized that those cases were probably caused by an iota-like toxin produced by *Clostridium spiroforme*, a toxin that cross-reacts with ITX by currently available toxin detection methods. To date, there are no reliable diagnostic criteria or gold standards for the diagnosis of *C. perfringens* type E-associated disease in animals [4,111].

### 5.2. General Mechanism of Action

The lipolysis-stimulated lipoprotein receptor (LSR) has been reported as a cellular receptor for Ib, which also mediates the toxin entering into host cells [112]. It has also been shown that entrance of ITX into host cells can involve cell-surface antigen CD44-associated endocytosis [113]. Once bound to its receptor, Ib assembles into heptamers which insert into the host plasma membrane forming functional channels, allowing the movement of ions, and the translocation and endocytosis of Ia (Figure 4) [114,115,116,117]. The enzymatic Ia component is also secreted as an inactive form that needs proteolytic removal of 9 to 11 N-terminal residues [110]. The Ia C-domain is responsible for the ADP-ribosylating activity of the toxin, which involves the covalent attachment of ADP-ribose onto an Arg at residue 177 of actin [118]. This leads to the depolymerization of actin filaments and increase of G-actin monomers [118,119]. Depolymerization of the actin cytoskeleton results in changes in cell morphology and disorganization of intercellular tight and basolateral junctions, leading to an increase permeability of cultured monolayers of intestinal cells [114]. Both ITX components internalize in targeted cells via a Rho-dependent, clathrin-independent pathway and reach endocytic vesicles [120]. A small proportion of Ib is recycled back to the plasma membrane, enhancing further uptake of Ia [121]. After endocytosis, Ia translocates from late endosomes into the cytoplasm where it exerts its ADP-ribosylating activity (Figure 4). This translocation requires the acidic environment present in late endosomes [122].

### 5.3. Mechanisms of Cell Death

It has been demonstrated that Ib by itself can induce cytotoxic activity, particularly in two human cell lines, namely A431 (epithelial carcinoma) and A549 (lung adenocarcinoma) [123]. Those cytotoxic effects involved marked cell swelling, mitochondrial dysfunction, ATP depletion and increased IP intake, all features consistent with necrosis. Even though activation of the pro-apoptotic molecules Bax and Bak and cytochrome C release was also observed, no activation of caspase-3 was detected and incubation of cells with the pan-caspase inhibitor Z-VAD-FMK did not protect cells from Ib-induced loss of viability. In that study, internalization of Ib was indicated as required for cell survival, suggesting a role for endocytosis in protecting cells against pore formation associated with Ib. In another study, however, ITX treatment of Vero cells induced caspase-3 activation after 12 to 15 h of incubation [124]. This delayed induction of apoptotic cell death was attributed to the cytosolic stability of the ADP-ribosylating Ia targeting actin (Figure 4).

## 6. *C. perfringens* Enterotoxin (CPE)

### 6.1. Toxin Genetics, Structure and Role in Disease

The *cpe* gene can be positioned on either the chromosome or on plasmids, and the expression of the toxin only occurs during sporulation [125,126]. CPE consists of a single polypeptide containing 319 amino acids [127]. The C-terminal half of the toxin lacks cytotoxic activity, but it mediates receptor binding, which involves the presence of several tyrosine residues located on the last 30 amino acids of CPE [128,129,130]. On the other hand, the N-terminal half is particularly important for cytotoxicity given its key role in CPE oligomerization and pore formation [131].

CPE is a pore-forming toxin produced during sporulation mainly by strains of *C. perfringens* type F [3] (Table 1), formerly known as CPE+ type A strains. However, CPE-producing strains belonging to C and D toxinotypes are also common [132]. Type E strains can carry a silent *cpe* gene [133] or produce a variant CPE [134]. Experimental and epidemiological evidence indicates CPE as the main virulence factor involved in *C. perfringens* type F-associated food poisoning of humans [125,127]. Molecular Koch’s postulates have been fulfilled in rabbits for CPE-producing type F strains [135]. *C. perfringens* type F strains are also involved in about 5–10% of all cases of antibiotic-associated diarrhea [136]. Symptoms of CPE-associated food poisoning include diarrhea and abdominal cramps, and they usually resolve spontaneously after a day or two [125]. However, under certain predisposing conditions, including constipation or fecal impaction, CPE can be lethal. It is thought that by reducing the effect of diarrhea in those situations, the toxin would prolong its contact with the intestine, facilitating the absorption and action on extra-intestinal organs [137,138]. This enterotoxemic effect is supported by studies performed in mouse-ligated intestinal loops, in which the inoculated CPE gained access to the circulation and caused hyperpotassemia resulting in death [139].

### 6.2. General Mechanism of Action

**Figure 5:**
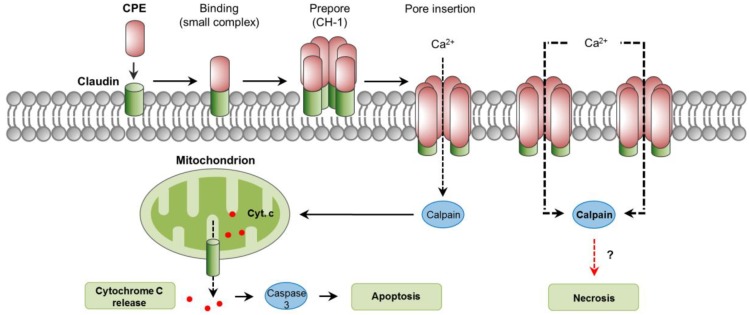
Intracellular pathways involved in *C. perfringens* enterotoxin (CPE) action. Claudins are cellular receptors for CPE. Initial CPE binding to claudins results in the formation of a small complex. Interaction of six small complexes lead to CPE oligomerization and formation of a pre-pore on the plasma membrane, a large complex named CH-1. Assembly of β-hairping loops from CPE into a β-barrel permits the insertion of a cation-permeating pore in the plasma membrane. An influx of Ca^2+^ occurs, which stimulates the activity of calpains, which in turn, leads to caspase-3 activation and apoptosis, or a mechanism of cell death with features of necrosis. A dashed red arrow represents what is concluded and/or suggested by the authors. A dashed black arrow shows direction of movement.

The cellular action of CPE has been previously described in detail [127]. The cellular receptors for CPE are several members of the claudin family, essential components of tight junctions located at the apical contact region between epithelial and endothelial cells [127]. Specifically, claudins 3, 4, 6, 8, and 14 are proven CPE receptors [140,141,142]. Initial binding of CPE to claudin receptors results in the formation of a “small complex” of ~90 kDa [143]. The interaction of several (about 6) of these small complexes leads to CPE oligomerization and formation of a prepore on the plasma membrane surface [144]. The result is a “large complex” of ~450 kDa, named CH-1, which contains the CPE hexamer, receptor and non-receptor claudins [143]. The formation of a cation-permeating pore is initiated by the assembly of β-hairpin loops from CPE into a β-barrel that rapidly inserts into the plasma membrane of the target cell [145]. This CPE pore allows the influx of calcium which is essential for CPE to cause cell death (Figure 5 [146,147,148]).

### 6.3. Mechanisms of Cell Death

When enterocyte-like Caco-2 cells are exposed to low CPE doses, a low number of pores are formed and a modest influx of calcium follows, resulting in low calpain activation and apoptosis, characterized by cytochrome C release and caspase-3 activation [148,149]. However, with higher CPE doses, the formation of many pores results in massive influx of calcium and strong calpain activation, leading to minimal caspase-3 activation and morphologic cellular changes consistent with necrosis [149]. Extended incubation times in vitro intensify those morphological changes, resulting in the exposure of the basolateral cell surface; this allows additional binding of CPE and the formation of an even larger complex of ~600 kDa named CH-2 that contains claudin and occludin proteins [143,150]. In small intestinal-loops made in mice and rabbits, CPE induced dose-dependent histologic damage characterized by severe villous shortening, desquamation, and changes in epithelial cells consistent with necrosis (e.g., pyknosis, karyorrhexis) [139,151]. Histologic damage also occurs in the rabbit colon [152]. In contrast to the in vitro findings previously commented, treatment of mice intestinal loops with CPE induced a dose- and time- dependent caspase-3 activation [153]. This activation, however, was not essential for the development of intestinal lesions, because the use of the pan-caspase inhibitor Q-VD-OPh did not protect from intestinal damage or enterotoxemic death [153]. The role of calpain activation and a potential involvement of necroptosis in CPE-associated disease have not been explored.

## 7. *C. perfringens* Necrotic Enteritis B-Like Toxin (NetB)

### 7.1. Toxin Genetics, Structure and Role in Disease

The *netB* gene is located on a 42 kb pathogenicity locus named NELoc1, present on large, conjugative plasmids [154,155,156,157]. Like CPB, NetB shares partial sequence similarity with a β-PFT, named alpha hemolysin, from *S. aureus* [158].

According to the updated toxinotyping scheme, NetB is produced by *C. perfringens* type G strains (Table 1) [3]. Molecular Koch’s postulates for this toxin have been fulfilled in chicken disease models [159]. Those studies confirmed that NetB is the primary virulence factor involved in the development of avian necrotic enteritis, which is also supported by strong epidemiological evidence [159,160,161].

### 7.2. General Mechanism of Action

Death of enterocytes in cases of avian necrotic enteritis has been described as a consequence of an initial destruction of the lamina propria, the extracellular matrix and intercellular junctions [162]. NetB forms heptameric, hydrophilic pores with a central diameter of approximately 26 Å [163]. The specific receptor for NetB, however, has not been identified [161].

### 7.3. Mechanisms of Cell Death

As for many other pore-forming toxins, NetB pores allow an influx of ions such as Na^+^, Cl^−^ and Ca^2+^ that may lead to osmotic cell lysis [160]. The toxin induces rounding and lysis of LMH cells, with subsequent LDH release [160]; although this is consistent with features of necrosis, specific pathways involved in cell death, to our knowledge, have not been explored for NetB action on the intestine.

## 8. Concluding Remarks

The body of knowledge about the molecular mechanisms of action of the main *C. perfringens* toxins has increased significantly over the past few years. It is interesting to observe, for example, how the hemolytic activity of CPA varies according to the affected host (horse vs sheep), and how ETX may induce intestinal lesions in goats in some cases of enterotoxemia, but rarely in other species. Host and cell susceptibility seem to be key elements for consideration when studying the pathogenesis of *C. perfringens* infections, and therefore the selection of either in vitro or animal models for *C. perfringens*-associated disease should address these variations. Since most *C. perfringens* toxins bind to and act on a receptor located on the plasma membrane of the host cell, this early step in the pathogenesis represents an evident therapeutic target for treating *C. perfringens*-associated diseases. In this regard, for instance, the therapeutic agent Mepacrine has been shown to protect enterocyte-like cells against CPE action in vitro by interfering with pore formation and insertion [164]. The potential effect of Mepacrine against CPE is currently being investigated in a mouse model. The final consequence in the action of most *C. perfringens* toxins is cell death. In the past few years significant progress has been made in understanding the complex intracellular pathways involved in this outcome by using different approaches. However, there are still many gaps in this knowledge, and dissecting the complex interaction between *C. perfringens* toxins and target cells may lead to the identification of additional pharmacological targets.

## Figures and Tables

**Table 1:** Revised classification system of *Clostridium perfringens* based on the production of six major toxins [3].

Type	Toxin Produced					
	α (CPA)	β (CPB)	ε (ETX)	ι (ITX)	CPE	NetB
A	+	−	−	−	−	−
B	+	+	+	−	−	−
C	+	+	−	−	+/−	−
D	+	−	+	−	+/−	−
E	+	−	−	+	+/−	−
F	+	−	−	−	+	−
G	+	−	−	−	−	+
